# Long non-coding RNAs in non-small cell lung cancer: implications for EGFR-TKI resistance

**DOI:** 10.3389/fgene.2023.1222059

**Published:** 2023-06-30

**Authors:** Detian Liu, Xiaolin Lu, Wentao Huang, Wei Zhuang

**Affiliations:** ^1^Department of Thoracic Surgery, Xiangya Hospital of Central South University, Changsha, Hunan, China; ^2^ The Second Clinical Medical College of Nanchang University, The Second Affiliated Hospital of Nanchang University, Nanchang, China

**Keywords:** long non-coding RNAs (lncRNA), epidermal growth factor receptor (EGFR), tyrosine kinase inhibitors (TKIs), drug resistance, non-small cell lung cancer (NSCLC)

## Abstract

Non-small cell lung cancer (NSCLC) is one of the most common types of malignant tumors as well as the leading cause of cancer-related deaths in the world. The application of epidermal growth factor receptor (EGFR)-tyrosine kinase inhibitors (TKIs) has dramatically improved the prognosis of NSCLC patients who harbor EGFR mutations. However, despite an excellent initial response, NSCLC inevitably becomes resistant to EGFR-TKIs, leading to irreversible disease progression. Hence, it is of great significance to shed light on the molecular mechanisms underlying the EGFR-TKI resistance in NSCLC. Long non-coding RNAs (lncRNAs) are critical gene modulators that are able to act as oncogenes or tumor suppressors that modulate tumorigenesis, invasion, and metastasis. Recently, extensive evidence demonstrates that lncRNAs also have a significant function in modulating EGFR-TKI resistance in NSCLC. In this review, we present a comprehensive summary of the lncRNAs involved in EGFR-TKI resistance in NSCLC and focus on their detailed mechanisms of action, including activation of alternative bypass signaling pathways, phenotypic transformation, intercellular communication in the tumor microenvironment, competing endogenous RNAs (ceRNAs) networks, and epigenetic modifications. In addition, we briefly discuss the limitations and the clinical implications of current lncRNAs research in this field.

## 1 Introduction

Lung cancer is the second most frequent type of malignant tumor that causes the highest cancer-related mortalities globally (Sung et al., 2021; Siegel et al., 2023). Lung cancer is categorized into non-small cell lung cancer (NSCLC) and small cell lung cancer (SCLC) according to the cell morphology. NSCLC, which primarily includes lung squamous cell carcinoma (LUSC), lung adenocarcinoma (LUAD), and large cell lung cancer, makes up almost 85% of lung cancer, while SCLC accounts for the remaining cases (Sung et al., 2021). Since NSCLC patients are not discovered and diagnosed until their symptoms are apparent in advanced stages, the prognosis of NSCLC patients is very poor, with the 5-year survival rate less than 20% (Choi and Mazzone, 2022; Ettinger et al., 2022).

EGFR mutations, which are revealed as drivers of NSCLC in 2004 (Lynch et al., 2004; Paez et al., 2004), are the most prevalent somatic genetic alterations in NSCLC that can be used as therapeutic targets. Approximately 20% of Caucasians and up to 40% of East Asians with NSCLC harbor EGFR mutations (Yatabe et al., 2015; Kobayashi and Mitsudomi, 2016). The advent of EGFR-TKIs has completely transformed the treatments for NSCLC, substantially prolonging the progression-free survival (PFS) as well as overall survival (OS) of NSCLC patients harboring EGFR mutations (Zhou et al., 2011). However, due to the development of EGFR-TKI resistance, most advanced NSCLC patients will inevitably experience disease progression around 10 months after treatment with EGFR-TKIs (Mok et al., 2009). The mechanisms of EGFR-TKI resistance in NSCLC include secondary or tertiary mutations of EGFR sites, upregulation of alternative bypass signaling pathways, phenotypic transformations, alterations in the tumor microenvironment, etc (Gomatou et al., 2023).

LncRNAs are non-coding RNAs (ncRNAs) that are more than 200 nt in length. Although lncRNAs were once regarded as transcriptional noise, they have a wide range of functions, interoperating with DNAs, RNAs, and proteins to regulate cellular genetic expression and diverse signaling pathways (Mercer et al., 2009). Many investigations have demonstrated that dysregulated lncRNAs are capable of acting as oncogenes or tumor suppressors to control the resistance to EGFR-TKIs in NSCLC through multiple mechanisms. Consequently, lncRNAs are likely to be new targets to overcome the resistance to EGFR-TKIs. In this review, we provide a brief overview of the biological occurrence and action mechanisms of lncRNAs. Furthermore, we synthetically summarize the various roles of lncRNAs in the mechanism of EGFR-TKI resistance. And finally, the current limitations and possible future trends in this field are also discussed.

## 2 An overview of lncRNAs

According to the Human Genome Project, about 2% of human genes can encode proteins, while over 90% of human genes are eventually transcribed into ncRNAs (Carninci et al., 2005; Birney et al., 2007; Ezkurdia et al., 2014). Initially, ncRNAs were regarded as junk fragments or transcriptional by-products with no practical role. However, new investigations have indicated that ncRNAs are critical for the biological function of cells (Prasanth and Spector, 2007; Statello et al., 2021). Based on their length, ncRNAs are divided in two groups: small non-coding RNAs (sncRNAs), which are less than 200 nt in length, and long non-coding RNAs (lncRNAs), whose lengths are more than 200 nt (Dinger et al., 2008). The majority of lncRNAs are transcribed from different genomic areas by RNA polymerase II as well as polymerase III (Dieci et al., 2007; Derrien et al., 2012). According to their transcriptional source areas, lncRNAs can be broadly classified into five types: intergenic lncRNAs, intronic lncRNAs, sense lncRNAs, antisense lncRNAs, and bidirectional lncRNAs. It is worth mentioning that some lncRNAs can form closed-loop structures, which are called circular RNAs (circRNAs) (St Laurent et al., 2015).

The action mechanisms of lncRNAs are complex and can be summarized into four types (Siegel et al., 2023): signals: lncRNAs are transcribed and act as signaling molecules to regulate the transcription of downstream genes under specific stimulation conditions (Sung et al., 2021); decoys: lncRNAs bind proteins or RNAs and block their actions (Choi and Mazzone, 2022); guides: lncRNAs incorporate and direct transcription factors to specific DNA sequences (Ettinger et al., 2022); scaffolds: lncRNAs are capable of serving as center platforms for multiple proteins to assemble into complexes (Rinn and Chang, 2012; Fang and Fullwood, 2016). Through these action modes, lncRNAs in the nucleus, cytoplasm, and exosomes can regulate gene expression on a variety of levels such as epigenetic, transcriptional as well as post-transcriptional levels, etc. As an example, in the nucleus, lncRNAs recruit histone modification enzymes to regulate histone modification (Li et al., 2016); in the cytoplasm, lncRNAs sponge miRNAs or form double strands with specific mRNAs to improve the stability of mRNAs (Rashid et al., 2016); and lncRNAs in exosomes can participate in intercellular communication (Zhang et al., 2017). Thus, as key genetic regulators, lncRNAs play comprehensive and pivotal roles in cellular physiological and pathological processes.

Emerging evidence suggests that lncRNAs are capable of acting as oncogenes or tumor suppressors to regulate oncogenesis, tumor progression, metastasis, recurrence, and drug resistance (Isin and Dalay, 2015). Additionally, the expression of lncRNAs exhibits high tissue specificity and tumor cell specificity (Yan et al., 2015). For instance, lncRNAs expression differs significantly in the EGFR TKI-resistant and EGFR TKI-sensitive NSCLC (Cheng et al., 2015a; Shi et al., 2020). Therefore, lncRNAs are probably valuable tumor diagnostic signatures as well as tumor treatment targets with great potential for clinical applications (Qi and Du, 2013), and this potential is even more prominent for exosomal lncRNAs (Tao et al., 2020; Han et al., 2021). Despite the huge amount of lncRNAs are already been found (Li et al., 2023), only a small fraction of lncRNAs have been elucidated regarding their mechanism of action and function. Moreover, the broad prospect of clinical applications based on lncRNAs has not yet become a reality. Thus, more research on lncRNAs, including the refinement of molecular mechanisms, the adjustment of research strategies, and the innovation of related technologies, is necessary.

## 3 LncRNAs and EGFR-TKI resistance in NSCLC

EGFR-TKIs are successful as first-line treatments for advanced NSCLC patients who harbor EGFR mutations. However, most patients develop drug resistance, which significantly diminishes the therapeutic efficacy of EGFR-TKIs treatments (Mok et al., 2009; Zhou et al., 2011). Cumulative evidence suggests that the mechanisms of EGFR-TKI resistance in NSCLC can be broadly divided into EGFR-dependent as well as EGFR-independent mechanisms (Gomatou et al., 2023). It is well known that the EGFR-dependent resistance mechanisms mainly refer to EGFR site mutations, among which T790M, a secondary mutation in EGFR site, is found to be the leading cause for resistance to the first- and second-generation EGFR-TKIs, such as gefitinib and erlotinib (Mok et al., 2017). And the EGFR C797S tertiary mutation is the most frequent EGFR-dependent resistance mechanism of third-generation EGFR-TKIs such as osimertinib (Oxnard et al., 2018). EGFR-independent resistance mechanisms, which can co-exist with EGFR site mutations, mainly include aberrant activation of alternative bypass signaling pathways, phenotypic transformation, alterations in the tumor microenvironment, etc. Aberrant activation in cellular signaling pathways, for example, the PI3K/AKT and classical MAPK pathways, dominates EGFR-independent resistance mechanisms, of which the common ones are mesenchymal-epithelial transition factor (MET) amplification, human epidermal growth factor receptor 2 (HER2) amplification, and oncogenic fusions (Liu et al., 2018).

Despite lacking the ability to encode proteins (Niazi and Valadkhan, 2012), lncRNAs are responsible for a wide range of biological processes. Aberrant lncRNAs expression is involved with many diseases, notably cancer (Bhan et al., 2017; Cao et al., 2022). In the setting of EGFR TKI-resistant NSCLC, lncRNAs are already been demonstrated to have significant roles (Wang Q. et al., 2020; Hu J. et al., 2021). There is not enough proof to support the role of lncRNAs in EGFR-dependent resistance mechanisms. However, numerous investigations have shown that lncRNAs have a broad control over EGFR-independent resistance mechanisms in NSCLC. After summarizing the large number of reported lncRNAs that are related to EGFR-TKI resistance, we have identified that in terms of activation of signaling pathways, lncRNAs mainly regulate three signaling pathways, which are the PI3K/AKT, classical MAPK, as well as JAK/STAT pathways; in terms of phenotypic transformation, epithelial to mesenchymal transition (EMT) is the main type of phenotypic transformation regulated by lncRNAs; in terms of the tumor microenvironment, exosomal lncRNAs affect EGFR-TKI response by participating in intercellular communication. Additionally, some lncRNAs modulate EGFR-TKI resistance via ceRNA networks and epigenetic modifications. In the following, we describe the above mechanisms through which lncRNAs mediate the EGFR-TKI resistance in NSCLC. The lncRNAs involved in EGFR-TKI resistance in NSCLC are listed in Table 1.

**TABLE 1:** LncRNAs involved in EGFR-TKI resistance of NSCLC.

Mechanism	LncRNA	Expression	Target/Signaling network	Function	Reference
alternative bypass signaling pathways	GAS5	↓	IGF-1R	gefitinib resistance	Dong et al. (2015)
	LINC01510	↑	MET	erlotinib resistance	Pal et al. (2022)
	UCA1	↑	miR-193a-3p/ERBB4	tumor-promoting	Nie et al. (2016)
	UCA1	↑	PI3K/AKT	gefitinib resistance	Cheng et al. (2015b)
	LINC01128	↓	miR-25-3p/PTEN	gefitinib resistance	Ding et al. (2022)
	LCETRL3	↑	TDP43/NOTCH1/PTEN	gefitinib resistance	Li et al. (2022)
	LCETRL4	↑	EIF2S1/PDK1	gefitinib resistance	Li et al. (2022)
	H19	↑	PTEN	gefitinib resistance	Zhou and Zhang (2020)
	H19	↓	PKM2	erlotinib resistance	Chen et al. (2020a)
	PCAT-1	↑	GSK3	gefitinib resistance	Wang et al. (2021a)
	MIR31HG	↑	MDM2/p53	gefitinib resistance	Wang et al. (2017b)
	CASC9	↑	miR-195-5p/FOXO3	gefitinib resistance	Bing et al. (2021)
	UCA1	↑	EZH2/p21	gefitinib resistance	Xu et al. (2020)
	HOTAIR	↑	EZH2/p21 and p16	gefitinib resistance	Li et al. (2021)
	LINC00460	↑	miR-769-5p/EGFR	gefitinib resistance	Ma et al. (2019)
	CRNDE	↑	eIF4A3/MUC1/EGFR	EGFR-TKI resistance	Takahashi et al. (2021)
	CASC9	↑	EZH2/DUSP1	gefitinib resistance	Chen et al. (2020b)
	LOC554202	↑	miR-31/RASA1 and FIH-1	gefitinib resistance	He et al. (2019)
	H19	↑	microRNA-107/NF1	tumor-promoting	Qian et al. (2018)
	LINC00460	↑	miR-149-5p/IL-6	gefitinib resistance	Nakano et al. (2020)
	TSLNC8	↓	IL-6/STAT3/HIF-1α	tumor-suppressor	Fan et al. (2019)
	TSLNC8	↓	EGFR/STAT3	osimertinib resistance	Zhou et al. (2020)
	PCAT6	↑	miR-326/IFNAR2	gefitinib resistance	Zheng et al. (2022)
	LINC01116	↑	IFI44	gefitinib resistance	Wang et al. (2020b)
	UCA1	↑	JAK/STAT	gefitinib resistance	Zhang et al. (2019)
	BLACAT1	↑	JAK/STAT	afatinib resistance	Shu et al. (2020)
phenotypic transformation	CASC8	↑	FOXM1	osimertinib resistance	Jiang et al. (2021)
	OSER1-AS1	↑	miR-612/FOXM1	gefitinib resistance	Shi et al. (2021)
	MALAT1	↑	ZEB1	gefitinib resistance	Feng et al. (2019)
	lnc-ABCA12-8	↑	FN1	gefitinib resistance	He et al. (2022)
	WT1-AS	↓	lncRNA UCA1	tumor-suppressor	Wan et al. (2021)
	WT1-AS	↓	miR-494-3p/PTEN	tumor-suppressor	Wu et al. (2021a)
	SNHG15	↑	miR-451/MDR-1	gefitinib resistance	Huang et al. (2020a)
exosomal lncRNAs in the tumor microenvironment	UCA1	↑	miR-143/FOSL2	gefitinib resistance	Chen et al. (2020c)
	H19	↑	hnRNPA2B1	gefitinib resistance	Lei et al. (2018)
	H19	↑	miR-615-3p/ATG7	erlotinib resistance	Pan and Zhou (2020)
	MSTRG.292666.16	↑	miR-6836-5p/MAPK8IP3	osimertinib resistance	Wan et al. (2022)
	SOX2-OT	↑	miR-627-3p/Smads	osimertinib resistance	Zhou et al. (2021)
	PCAT6	↑	miR-326/KLF1	tumor-promoting	Chen et al. (2022)
	lnc-MZT2A-5:1	↑	Undefined	tumor-promoting	Song et al. (2021)
other mechanisms	PCAT6	↑	miR-330-5p	tumor-promoting	Cui et al. (2018)
	SNHG14	↑	miR-206-3p/ABCB1	gefitinib resistance	Wu et al. (2019)
	HOST2	↑	miRNA-621/SYF2	gefitinib resistance	Chen et al. (2019)
	lnc-TMEM132D-AS1	↑	miR-766-5p/ENTPD1	osimertinib resistance	Wang et al. (2023)
	MIAT	↑	Dnmt3a/miR-34a	gefitinib resistance	Fu et al. (2018)
	FTH1P3	↑	LSD1/TIMP3	gefitinib resistance	Zheng et al. (2020a)
	HAS2-AS1	↑	LSD1/EphB3	gefitinib resistance	Sun et al. (2020)
	LINC00665	↑	EZH2	gefitinib resistance	Liu et al. (2019)
	SNHG17	↑	EZH2/LATS2	gefitinib resistance	Zhang et al. (2022)
	LINC00969	↑	EZH2 and METTL3/NLRP3	gefitinib resistance	Dai et al. (2023)

## 4 Modulation of alternative bypass signaling pathways

### 4.1 The PI3K/AKT signaling pathway

**FIGURE 1:**
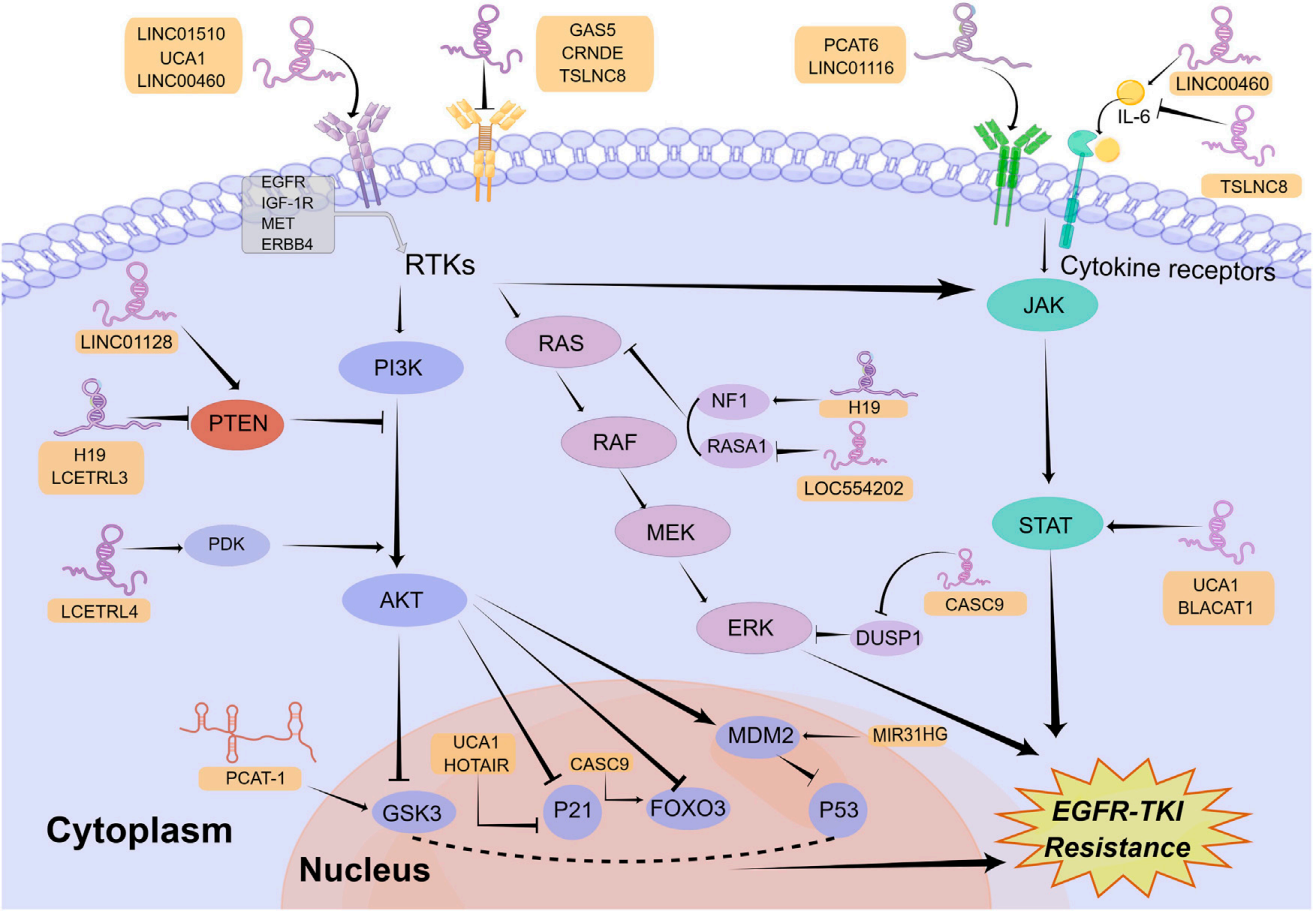
Mechanisms of lncRNAs involved in EGFR-TKI resistance in NSCLC by modulating signaling pathways. LncRNAs can regulate multiple signaling pathways to affect EGFR-TKI resistance in NSCLC, including the PI3K/AKT, classical MAPK, and JAK/STAT signaling pathways. Mechanistically, lncRNAs activate or inactivate these pathways by regulating their upstream receptors, regulatory factors, and downstream molecules. The Figure was drawn by Figdraw.

Survival signals induced by multiple receptors are mediated mainly through the PI3K/AKT signaling pathway, which has pivotal functions for the regulation of cellular growth, cell cycle progression, and cell survival (Fresno Vara et al., 2004). Abnormal upregulation of the PI3K/AKT signaling pathway facilitates the resistance to EGFR-TKIs in NSCLC (Liu et al., 2018). Extensive investigations have already documented that the upstream receptors, regulatory factors, and downstream molecules of this pathway can be regulated by lncRNAs to modulate EGFR-TKI resistance in NSCLC (Figure 1).

A number of receptors of the PI3K/AKT signaling pathway, such as insulin-like growth factor 1 receptor (IGF-1R), MET and EGFR, are regulated by lncRNAs to affect EGFR-TKI response in NSCLC. For example, in an investigation by Dong and collaborators, lncRNA GAS5 was revealed to be lowly expressed in gefitinib-resistant NSCLC cell lines. Overexpression of GAS5 reversed gefitinib resistance *in vitro* and *in vivo* through downregulating IGF-1R expression and inhibiting PI3K/AKT pathway (Dong et al., 2015). In another recent study, investigators found that loss of KMT5C in erlotinib-resistant cell lines resulted in enhanced transcription of lncRNA LINC01510, which could lead to upregulation of MET expression, activation of PI3K/AKT signaling pathway, and ultimately erlotinib resistance (Pal et al., 2022). The highlight in this study is the elucidation of the upstream mechanisms of LINC01510 dysregulation. In addition, since MET amplification is the most common EGFR-independent resistance mechanism (Qin et al., 2023), KMT5C/LINC01510/MET axis is of great importance in overcoming EGFR-TKI resistance and might become a potential target. In addition to IGF-1R and MET, lncRNAs also regulate other receptors in the PI3K/AKT pathway. LncRNA TRPM2-AS was reported to facilitate NSCLC cell proliferation, migration, and invasion by upregulation of EGFR and activation of the PI3K/AKT pathway via functioning as a ceRNA of miR-138-5p (Cui et al., 2020). However, whether lncRNA TRPM2-AS promotes EGFR-TKI resistance in NSCLC is still unclear, which urgently requires further exploration. Similarly, lncRNA UCA1 functioned as the ceRNA of miR-193a-3p to upregulate ERBB4 (Nie et al., 2016), which can activate the PI3K/AKT pathway (Yu et al., 2015). Moreover, UCA1 was revealed to induce EGFR-TKI resistance in non-T790M NSCLC cells via activating the AKT/mTOR pathway in another study (Cheng et al., 2015b). Thus, the miR-193a-3p/ERBB4 axis may be the mechanism by which lncRNA UCA1 leads to EGFR-TKI resistance in NSCLC.

Among numerous regulatory factors in the PI3K/AKT pathway, Phosphatase and Tensin Homolog (PTEN), one well-known inhibitor in this pathway (Kim et al., 2010), is modulated by lncRNAs to affect EGFR-TKIs response in NSCLC. Mainly through bioinformatics analysis, Ding and collaborators discovered LINC01128 was low expressed in gefitinib-resistant NSCLC cells and LINC01128/miR-25-3p/PTEN pathway was likely to enhance the resistance to EGFR-TKIs in NSCLC through mediating the PI3K/AKT pathway (Ding et al., 2022). However, due to the lack of sufficient experiments in this study, additional *in vitro* and *in vivo* experiments will be needed for the confirmation of their conclusions. In a recent investigation, Li et al. first identified lncRNAs LCETRL3 and LCETRL4 as new oncogenic genes that were highly expressed in NSCLC tissues and can reduce gefitinib efficiency against NSCLC *in vitro* and *in vivo*. Mechanistically, LCETRL3 activates NOTCH1-PTEN-AKT signaling by stabilizing TDP43 and LCETRL4 activates PDK1-AKT signaling by stabilizing EIF2S1 (Li et al., 2022). It was revealed that lncRNA H19 was upregulated in lung cancer cells and H19 knockdown augmented the gefitinib sensitivity by enhancing PTEN and PDCD4 and decreasing NFIB (Zhou and Zhang, 2020). Paradoxically, in another study, H19 was identified as significantly downregulated in clinical samples and *in vitro* models of resistance to EGFR-TKIs. H19 knockdown contributed to erlotinib resistance by upregulating PKM2 and activating the AKT pathway (Wang C. et al., 2017; Chen C. et al., 2020). Consequently, the exact effect of H19 within the mechanism of EGFR-TKI resistance in NSCLC still needs to be further explored.

In addition to modulating upstream receptors and regulatory factors, lncRNAs also regulate downstream molecules of the PI3K/AKT pathway, such as glycogen synthase kinase-3 (GSK3), mouse double minute 2 homolog (MDM2), FOXO3 and P21, to influence EGFR-TKI resistance in NSCLC. For example, in a study by Wang et al., lncRNA PCAT-1 was found to be highly expressed in EGFR TKI-resistant NSCLC and its knock-down inhibited the phosphorylation of AKT and GSK3, thus improving gefitinib sensitivity in NSCLC (Wang S. et al., 2021). But the more detailed mechanism of GSK3 regulation by PCAT-1 is unclear and requires further exploration. Similarly, the knockdown of lncRNA MIR31HG inhibited the PI3K/AKT signaling pathway and MDM2, thereby increasing p53 expression and enhancing gefitinib sensitivity in PC9-R cells (Wang B. et al., 2017). Interestingly, Bing et al. identified the lncRNA CASC9/miR-195-5p/FOXO3 positive feed-back loops that can enhance gefitinib resistance in NSCLC (Bing et al., 2021). In addition, both lncRNAs UCA1 and HOTAIR promoted gefitinib resistance in NSCLC through epigenetically silencing p21 expression by interacting with EZH2, a methyltransferase responsible for the trimethylation of H3K27 (Xu et al., 2020; Li et al., 2021). According to the large number of lncRNAs reported above, the regulation of the PI3K/AKT signaling pathway by lncRNAs has a significant impact in EGFR-TKI resistance.

### 4.2 The classical MAPK signaling pathway

The classical MAPK signaling pathway, which is generally called the RAS/MEK/ERK pathway, is one of the key signaling pathways downstream of EGFR that modulates cancer growth, survival, migration, and angiogenesis (Martinelli et al., 2017). It has already been shown that the dysregulation of this pathway was related to EGFR-TKI resistance in NSCLC (Coco et al., 2015). In a similar way to the PI3K/AKT signaling pathway, lncRNAs can activate or inhibit the classical MAPK signaling pathway by regulating its upstream receptors and regulatory factors (Figure 1).

EGFR is the primary upstream receptor in the classical MAPK pathway that is regulated through lncRNAs. For example, lncRNA LINC00460 was demonstrated to promote gefitinib resistance in NSCLC via sponging miR-769-5p and facilitating EGFR expression (Ma et al., 2019). Nevertheless, lncRNA CRNDE downregulated eIF4A3, mucin 1 (MUC1), as well as p-EGFR, thereby increasing EGFR-TKI resistance (Takahashi et al., 2021). Therefore, the relationship between EGFR expression and EGFR-TKI resistance requires further investigation. Furthermore, lncRNA DUXAP9-206 was found to interact with Cbl‐b, one of the E3 ubiquitin ligases, to partially reduce EGFR degradation and activate the RAS/MEK/ERK and PI3K/AKT pathways, resulting in a degree of promotion of the malignant phenotype in NSCLC cells (Zhu et al., 2019). It indicates that DUXAP9-206 may be involved in EGFR-TKI resistance. In addition to activating the PI3K/AKT signaling pathway, as mentioned above, lncRNA LINC01510 can activate the classical MAPK signaling pathway by upregulating MET to promote EGFR-TKI resistance (Pal et al., 2022). Moreover, other lncRNAs, such as Linc00284, FAM83A-AS1, and LINC00857, were revealed to mediate lung cancer progression through the regulation of MET (Su et al., 2020; Sheng et al., 2021; Zhao et al., 2022), but their roles in EGFR-TKI resistance need to be further explored.

The modulation of regulators in the classical MAPK pathway, including DUSP1 as well as RASA, by lncRNAs contributes to EGFR-TKI resistance. Mainly through bioinformatics analysis, Ma et al. revealed that lncRNA CASC9 mediated EGFR-TKI resistance via interacting with some protein-coding genes (PCGs) (Ma et al., 2017). In a subsequent study, Chen et al. revealed that CASC9 epigenetically silenced DUSP1 and activated the ERK pathway by recruiting EZH2, resulting in enhancing gefitinib resistance in NSCLC (Chen Z. et al., 2020). Considering that the CASC9/miR-195-5p/FOXO3 regenerative feed-back loops described above can also enhance gefitinib resistance (Bing et al., 2021), CASC9 may be a target with great promise for surmounting EGFR-TKI resistance in NSCLC. Moreover, lncRNA LOC554202 promoted gefitinib resistance by upregulating the expression of miR-31, which can directly repress RASA1 and Hypoxia Inducible Factor 1 Subunit Alpha Inhibitor (FIH-1) expression, resulting in partial upregulation of the classical MAPK and PI3K/AKT pathways (He et al., 2019). Similar to DUSP1 and RASA1, as a negative regulatory factor in the classical MAPK pathway, NF1 is also capable of affecting EGFR-TKI resistance in NSCLC (de Bruin et al., 2014). One investigation has reported lncRNA H19 increased NF1 by competitively combining with microRNA-107, thus promoting NSCLC progression (Qian et al., 2018). In theory, the upregulation of NF1 could inhibit the classical MAPK signaling pathway, thereby inhibiting tumor development, which is contrary to the results of this study. Therefore, the mechanism by which H19 regulates NF1 and the classical MAPK signaling pathway needs to be further explored. In addition, whether H19 promotes EGFR-TKI resistance in NSCLC by regulating NF1 is also unknown and needs further investigation.

### 4.3 The JAK/STAT signaling pathway

The JAK/STAT signaling pathway, which is mainly composed of ligands, receptors, JAKs and STATs, plays a crucial role in mediating immune adaptation, tissue repair, inflammatory response, and cell apoptosis (Owen et al., 2019). The abnormal upregulation of this pathway is related to tumor progression as well as drug resistance (Jin, 2020; Hu X. et al., 2021). LncRNAs are able to mediate EGFR-TKI resistance in NSCLC via impacting the ligands, receptors, and regulatory factors in the JAK/STAT pathway (Figure 1).

More than 50 cytokines have been identified in the JAK/STAT signaling pathway (Morris et al., 2018). Interleukin 6 (IL-6), an important cytokine associated with chronic inflammatory diseases, was engaged in the progression of lung cancer (Gao et al., 2007). Researchers discovered that lncRNA LINC00460 can competitively bind with miR-149-5p to facilitate IL-6 expression, thus promoting gefitinib resistance of NSCLC cells. In mechanism, IL-6 can activate the JAK/STAT and PI3K/AKT pathways, thereby inducing an EMT-like phenotype, which is one of the mechanisms responsible for EGFR-TKI resistance in NSCLC (Nakano et al., 2020). Similarly, lncRNA TSLNC8 was downregulated in NSCLC and its over-expression suppressed the development of NSCLC by modulating the IL-6/STAT3/HIF-1α pathway (Fan et al., 2019). It is noteworthy that in another study, upregulation of TSLNC8 also markedly augmented the antitumor activity of osimertinib on NSCLC by repressing the EFGR/STAT3 signaling pathway (Zhou et al., 2020). Therefore, TSLNC8 is able to impact the JAK/STAT pathway not only by regulating IL-6 but also by EGFR, thus mediating the development as well as EGFR-TKI resistance of NSCLC. In addition, lncRNA PCAT6 was revealed to augment gefitinib resistance by serving as a competitive endogenous RNA for miR-326 in increasing interferon-alpha receptor 2 (IFNAR2) expression, an upstream receptor in the JAK/STAT pathway (Zheng et al., 2022).

Similar to the PI3K/AKT and classical MAPK signaling pathways, lncRNAs are also able to modulate the JAK/STAT pathway via affecting its regulatory factors. For example, interferon-induced protein 44 (IFI44), an upstream regulator in the JAK/STAT signaling pathway, was found to be inhibited by LINC01116 to promote gefitinib resistance in NSCLC (Wang H. et al., 2020). In addition, both lncRNA UCA1 and lncRNA BLACAT1 have been reported to mediate EGFR-TKI resistance of NSCLC cells through targeting the JAK/STAT signaling pathway (Zhang et al., 2019; Shu et al., 2020). The fundamental mechanisms through which these lncRNAs modulate the JAK/STAT signaling pathway, however, are still unknown and require additional investigation.

## 5 Modulation of phenotypic transformation

Phenotype transformations, including EMT, transformations from LUAD to LUSC as well as NSCLC to SCLC, are considered to be essential mechanisms of EGFR-TKI resistance in NSCLC (Rubin et al., 2020; Shaurova et al., 2020). EMT was found to be a reversible procedure in which cancer cells lose epithelial features as well as gain mesenchymal phenotypes, resulting in the acquisition of invasiveness, metastasis, and drug resistance (Witta et al., 2006; Singh and Settleman, 2010). The molecular mechanisms underlying EMT are not well studied. Current evidence suggests that this process is accompanied by abnormal expression of a wide range of molecules, including reduced expression of epithelial markers, for example E-cadherin, as well as upregulation of mesenchymal markers, for example vimentin (Sinha et al., 2020). Furthermore, alterations of multiple transcription factors (Yuan et al., 2019) and cellular signaling pathways, such as transforming growth factor-β (TGF-β), Wnt/β-catenin and PI3K/AKT signaling pathways, were found to be involved in EMT (Xu et al., 2015; Hao et al., 2019; Liu L. et al., 2020). According to recent studies, some lncRNAs associated with EGFR-TKI resistance can mediate EMT mainly by regulating transcription factors and some signaling pathways. In addition, SCLC transformation and LUSC transformation are also implicated with EGFR-TKI resistance (Shaurova et al., 2020). Nevertheless, the mechanism by which lncRNAs in regulating SCLC and LUSC transformation is ambiguous and urgently needs to be further explored.

It is revealed that silencing lncRNA CASC8 promoted osimertinib sensitivity in NSCLC cells through downregulation of Forkhead box M1 (FOXM1) (Jiang et al., 2021), one of the critical transcription factors for EMT (Balli et al., 2013; Wei et al., 2015). Researchers in this study speculated that CASC8 acted as the competitive endogenous RNA for miR-671-5P to enhance the transcription of FOXM1. However, the exact mechanism is not explored and needs further validation. Similarly, lncRNA OSER1-AS1 served as a sponge for miR-612 to enhance FOXM1 transcription, resulting in promoting gefitinib resistance (Shi et al., 2021). Zinc finger E-box binding homeobox 1 (ZEB1) has also been proven to be a transcription factor that can induce EMT in carcinoma cells (Zhang et al., 2015). LncRNA MALAT1 can promote gefitinib resistance through the miR-200a/ZEB1 axis (Feng et al., 2019). In another study, it was demonstrated that MALAT1 can promote EMT by sponging miR-124 (Wu et al., 2018). Therefore, the MALAT1/ZEB1 pathway may affect EGFR-TKI resistance by promoting EMT. LncRNA linc00673 can upregulate ZEB1 by sponging miR-150-5p to promote EMT (Lu et al., 2017). Nevertheless, the association of linc00673 with EGFR-TKI resistance needs to be further studied. Furthermore, through *in vitro* and *in vivo* experiments, lnc-ABCA12-8 was revealed to promote gefitinib resistance in NSCLC via interacting with alternative splicing factor/splicing factor 2 (ASF/SF2) and enhancing the expression of IIICS region of fibronectin 1 (FN1) (He et al., 2022), an important marker of EMT (Liu et al., 2020b).

In terms of signaling pathways, lncRNA LINC00460 was shown to serve as a sponge of miR-149-5p to activate the JAK/STAT and PI3K/AKT signaling pathways to induce EMT, thus promoting EGFR-TKI resistance in NSCLC (Nakano et al., 2020). Researchers revealed the low expression of lncRNA WT1-AS rendered lncRNA UCA1 highly expressed, thereby promoting EMT (Wan et al., 2021). Interestingly, WT1-AS also functioned as a competitive endogenous RNA of miR-494-3p to activate the PI3K/AKT pathway, thus facilitating the proliferation, migration, and invasion in NSCLC (Wu C. et al., 2021). Moreover, UCA1 was revealed to regulate gefitinib resistance through activating the PI3K/AKT pathway and mediating EMT (Cheng et al., 2015b). For this reason, the mechanism of the regulatory relationship between WT1-AS and UCA1 in EGFR-TKI resistance in NSCLC deserves further exploration. Additionally, NOTCH-1, the primary upstream receptor of NOTCH signaling pathway that regulates EMT (Xie et al., 2013), conferred gefitinib resistance of NSCLC via the lncRNA SNHG15/miR-451/ZEB1 feedback loop (Huang J. et al., 2020). Moreover, lncRNA XIST and HCP5 can promote EMT mediated by TGF-β through modulating miR-367/miR-141-ZEB2 or miR-203/SNAI axis, respectively (Li et al., 2018; Jiang et al., 2019). Nevertheless, it is not clear whether lncRNA XIST and HCP5 can mediate EGFR-TKI resistance of NSCLC via promoting EMT, which warrants further investigation.

## 6 Exosomal lncRNAs in the tumor microenvironment

**FIGURE 2:**
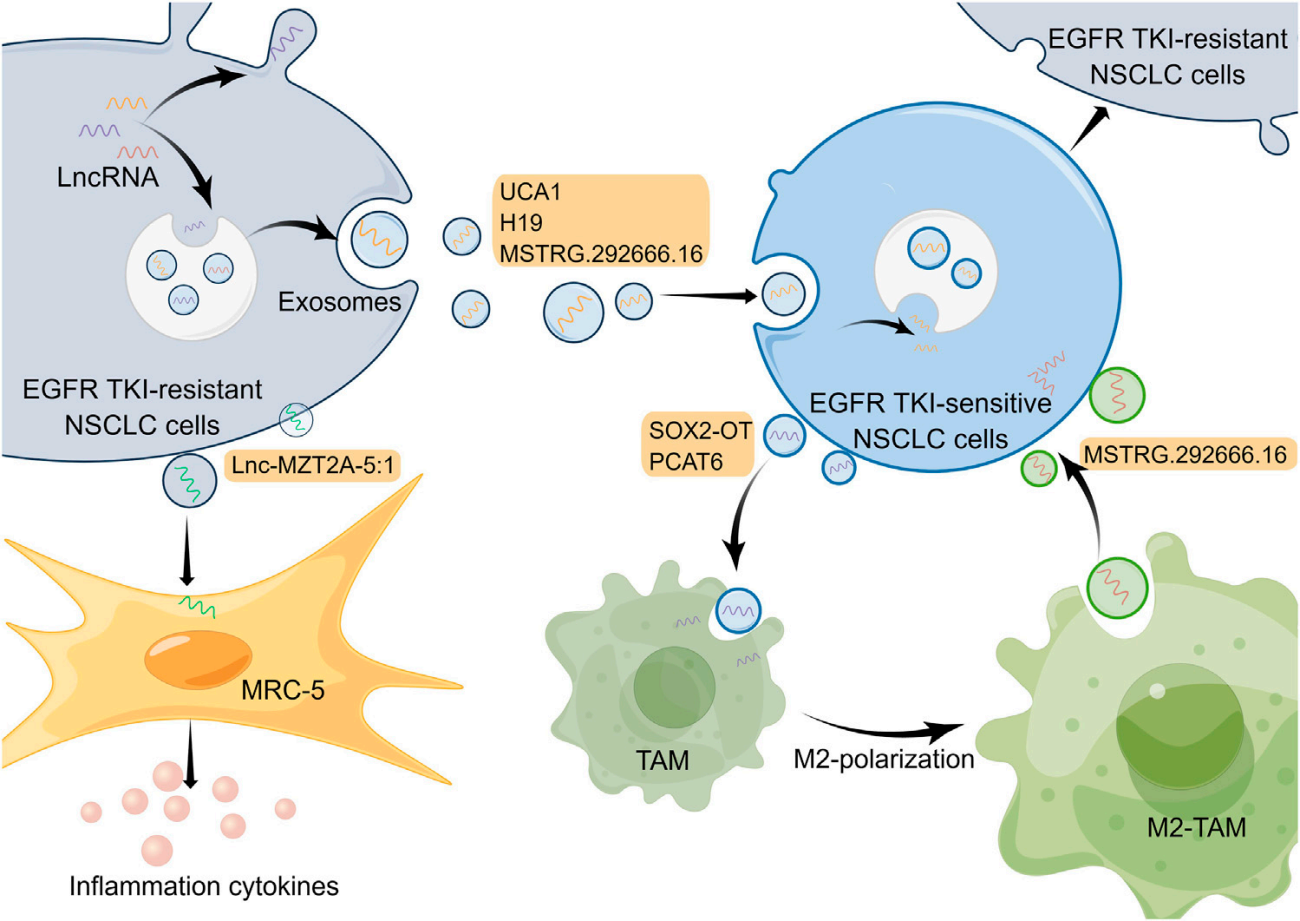
Mechanisms of lncRNAs involved in EGFR-TKI resistance in NSCLC by modulating tumor microenvironment. LncRNAs in cells can be transported and secreted into the tumor microenvironment via exosomes to participate in intercellular communication. And exosomal lncRNAs are able to affect EGFR-TKI resistance in NSCLC by regulating EGFR TKI-sensitive NSCLC cells and other non-tumor cells. The Figure was drawn by Figdraw.

Tumor formation results from the interaction of tumor cells with the extracellular matrix, tumor vasculature, and a variety of immune cells, so the development, metastasis, and drug resistance of tumor depend not only on genetic alterations within the tumor cells but also on the external tumor microenvironment (Junttila and de Sauvage, 2013). The tumor microenvironment, mainly including the vascular network, catabolic cancer-associated fibroblasts (CAFs), immune-related cells, as well as extracellular matrix (Klemm and Joyce, 2015), has a pivotal function in EGFR-TKI resistance in NSCLC (Jia et al., 2019; Dzul Keflee et al., 2022). Currently, many studies on the tumor microenvironment of lung cancer focus on immune cells and immunotherapy; nevertheless, the roles of exosomes from the tumor microenvironment for resistance to EGFR-TKIs in NSCLC are also significant (Liu et al., 2020c; Yuan et al., 2022). Released by eukaryotic cells, exosomes, a type of extracellular vesicles that mainly contain proteins, lipids and genetic materials, play essential roles in the intracellular communication network in the tumor microenvironment (Mostafazadeh et al., 2021). Substantial evidence indicates that lncRNAs in tumor cells or other cells can be transported as well as secreted into the tumor microenvironment via exosomes to regulate cell function and promote drug resistance (Pathania and Challagundla, 2021). Exosomal lncRNAs in the tumor microenvironment affect EGFR-TKI resistance of NSCLC mainly by regulating EGFR TKI-sensitive NSCLC cells or other non-tumor cells (Figure 2).

### 6.1 Regulation of EGFR TKI-sensitive NSCLC cells

Exosomes in the tumor microenvironment can be taken up by adjacent EGFR TKI-sensitive NSCLC cells, which results in NSCLC cells becoming resistant to EGFR-TKIs (Wu S. et al., 2021). For example, lncRNA UCA1 can be delivered to gefitinib-sensitive recipient cells by exosomes isolated from gefitinib-resistant cell lines. And it promoted gefitinib resistance in NSCLC *in vitro* and *in vivo* through functioning as a ceRNA for miR-143 and modulating FOSL2 expression (Chen X. et al., 2020). It has been reported that hnRNPA2B1, an RNA-binding protein that regulates RNA loading into exosomes, mediated the packaging of lncRNA H19 into exosomes, which promoted gefitinib resistance in NSCLC cells (Lei et al., 2018). Additionally, another research has demonstrated that exosomal lncRNA H19 could enhance erlotinib resistance in NSCLC cells through the regulation of miR-615-3p/ATG7 axis (Pan and Zhou, 2020), indicating that exosomal lncRNA H19 may be a potential treatment target for NSCLC. Moreover, Deng et al. discovered exosomal lncRNA MSTRG.292666.16 was able to be uptaken by osimertinib-sensitive NSCLC cells, which led to these cells becoming resistant to osimertinib (Deng et al., 2020). However, given that this study only used one cell line for *in vitro* experiments, the mechanism by which this exosomal lncRNA modulates EGFR-TKI resistance needs to be explored in more NSCLC cell lines and *in vivo* experiments. In a subsequent study, investigators isolated exosomal lncRNA MSTRG.292666.16 from M2 type tumor-associated macrophages (TAMs) and demonstrated that it could promote osimertinib resistance of NSCLC through modulating miR-6836-5p/MAPK8IP3 axis (Wan et al., 2022). Thus, exosomal lncRNA MSTRG.292666.16 may be used as a promising therapeutic target to overcome osimertinib resistance in NSCLC. These studies illustrate that exosomal lncRNAs associated with EGFR-TKI resistance can be derived not only from EGFR TKI-resistant NSCLC cells but also from non-tumor cells, such as M2 type TAMs, which fully exemplifies the complexity of cellular communication in the tumor microenvironment.

### 6.2 Regulation of other non-tumor cells

Besides regulating tumor cells, exosomes in the tumor microenvironment can act on other non-tumor cells and, in turn, influence the drug resistance in tumor cells. For instance, in an investigation conducted by Zhou and collaborators, exosomal lncRNA SOX2 overlapping transcript (SOX2-OT) was delivered from NSCLC cell line H1975 to THP-1 cells and it was able to enhance macrophage M2 polarization as well as inhibit macrophage M1 polarization by regulating miR-627-3p/Smad axis, thereby promoting EGFR-TKI resistance (Zhou et al., 2021). Similarly, exosomal lncRNA PCAT6 derived from NSCLC cells also could promote macrophage M2 polarization, which enhances metastasis and EMT processes in NSCLC through modulating miR-326/KLF1 pathway (Chen et al., 2022). In addition, the phenomenon of macrophage M2 polarization in the tumor microenvironment is able to promote the EGFR-TKI resistance of NSCLC cells (Xiao et al., 2020). So exosomal lncRNAs may serve as targets to inhibit macrophage M2 polarization to suppress NSCLC. Furthermore, exosomal lnc-MZT2A-5:1 from AZD9291-resistant NSCLC cells was significantly upregulated compared with wild-type NSCLC cells, and it was able to enhance the activation of lung fibroblasts (MRC-5 cells) (Song et al., 2021). Nevertheless, whether and how activated MRC-5 cells can further influence the tumor microenvironment and EGFR-TKI resistance of NSCLC urgently requires in-depth exploration.

## 7 LncRNAs and other mechanisms

### 7.1 CeRNA networks

The intricate interplay among diverse RNA species critically contributes to the regulation of cellular functions. Different RNA transcripts that have the same miRNA response elements (MREs) and constitute a competitive relationship can act as competitive endogenous RNAs (ceRNAs), the so-called miRNA sponges (Tay et al., 2014). In recent years, there is accumulating evidence suggesting that lncRNAs and their constituent ceRNA networks are implicated with EGFR-TKI resistance of NSCLC (Kong et al., 2020; Wang T. et al., 2021). Many lncRNAs that act as ceRNAs to regulate EGFR-TKI resistance in NSCLC have been mentioned above. However, because ceRNA networks are unique and important for drug resistance, there are some points to be emphasized and elaborated. LncRNA PCAT6 has been reported to enhance gefitinib resistance of NSCLC through modulation of miR-326/IFNAR2 pathway (Zheng et al., 2022). Another study found PCAT6 promoted EMT process in NSCLC through miR-326/KLF1 pathway (Chen et al., 2022). Moreover, PCAT6 can enhance NSCLC proliferation, migration and invasion via acting as ceRNA of miR-330-5p (Cui et al., 2018). Accordingly, one lncRNA can competitively bind to different miRNAs, and one miRNA can also target different mRNAs, illustrating that ceRNA networks are heterogeneous.

In the field of lncRNAs and ceRNA networks research, bioinformatics prediction tools are used to predict and construct lncRNA-miRNA-mRNA Networks (Rincón-Riveros et al., 2021), which could subsequently be validated with dual luciferase reporter or RNA immunoprecipitation assays (Huang et al., 2019). For example, Wu et al. predicted and validated that lncRNA SNHG14 could sponge to miR-206-3p through online software starbase v2.0 and luciferase reporter analysis. Then similarly, they predicted and validated ABCB1, a member in the ATP-binding cassette (ABC) transporter family, as the target of miR-206-3p. Eventually, they demonstrated that SNHG14 promoted gefitinib resistance of NSCLC via the regulation of miR-206-3p/ABCB1 axis (Wu et al., 2019). In similar ways, it is proved lncRNA human ovarian cancer-specific transcript 2 (HOST2) facilitated gefitinib resistance via functioning as ceRNA for miRNA-621 to upregulate SYF2, a cell cycle-associated protein (Chen et al., 2019). In addition, CCAT1 and lnc-TMEM132D-AS1 were demonstrated to regulate EGFR-TKI resistance in NSCLC through their corresponding ceRNA networks (Jin et al., 2020; Wang et al., 2023). This evidence demonstrates the significance of bioinformatics prediction tools for ceRNA networks research. However, due to the wide variability in the timing, space, as well as the abundance of expression of diverse RNA species in cells, the prediction strategies of bioinformatic tools need to be continuously refined (Ebert and Sharp, 2010; Tay et al., 2014).

### 7.2 Epigenetic modifications

Cancer is both a multi-genic as well as an epigenetic disease, as there are not only genetic mutations but also a great number of epigenetic alterations in tumor cells (Iacobuzio-Donahue, 2009). Epigenetic modifications, for example, DNA methylation and histone modification, are critical in tumor growth, metastasis, and drug resistance (Gao et al., 2017; Bajbouj et al., 2021). LncRNAs can affect EGFR-TKI resistance in NSCLC through epigenetic modifications. For instance, lncRNAs MIAT recruited Dnmt3a, a DNA methyltransferase, to methylate the miR-34a promotor, leading to silencing miR-34a expression and finally conferring gefitinib resistance through the PI3K/AKT signaling pathway (Fu et al., 2018). Furthermore, lncRNA ferritin heavy chain 1 pseudogene 3 (FTH1P3) accelerated gefitinib resistance of NSCLC by recruiting lysine-specific demethylase 1 (LSD1), a histone-modifying enzyme, and epigenetically inhibiting the tissue inhibitor of metalloproteinase 3 (TIMP3) (Zheng G. et al., 2020). Likewise, lncRNA HAS2-AS1 can recruit LSD1 to the EphB3 promoter area and repress EphB3 transcription, thus promoting gefitinib resistance (Sun et al., 2020). EZH2, one of the key subunits in the polycomb repressive complex 2 (PRC2) complex, is the histone methyltransferase that can catalyze the trimethylation of H3K27me3 (Chase and Cross, 2011). It was reported that LINC00665 can mediate gefitinib resistance in NSCLC via interacting with EZH2 and activating the PI3K/AKT pathway (Geng et al., 2015; Liu et al., 2019). In addition, lncRNA UCA1 and HOTAIR are also able to facilitate gefitinib resistance through modification of EZH2 (Xu et al., 2020; Li et al., 2021). Therefore, EZH2 modified by lncRNAs plays a great role in EGFR-TKI resistance in NSCLC and is a possible therapeutic target for overcoming EGFR-TKI resistance in NSCLC, which deserves further exploration.

### 7.3 Epitranscriptomic modifications

In addition to epigenetic modifications, RNA modifications, called epitranscriptomic modifications, are also one of the mechanisms by which lncRNAs regulate EGFR-TKI resistance in NSCLC. Epitranscriptomic modifications modulate the function of mRNAs or ncRNAs by adding functional groups to them, of which methylation modifications are the most common type, such as N6-methyladenosine (m6A), 5-methylcytosine (m5C), N1-methyladenosine (m1A), etc (Barbieri and Kouzarides, 2020). Epitranscriptomic modifications can act not only as the upstream mechanism for the dysregulation of lncRNAs but also as the downstream mechanism for lncRNAs to function. For example, Zhang et al. found that METTL3-mediated m6A modification upregulated lncRNA SNHG17 by stabilizing its transcription, thereby promoting gefitinib resistance in NSCLC via the EZH2/LATS2 pathway (Zhang et al., 2022). LINC00969 was found to interact with both EZH2 and METTL3 to regulate histone methylation levels in the promoter region of NLRP3 and the m6A levels of NLRP3 mRNA to inhibit the expression of NLRP3, which promotes gefitinib resistance (Dai et al., 2023). In addition, lncRNA DGUOK-AS1 can promote the malignant phenotype of NSCLC by regulating the m6A modification of TRPM7 (Feng et al., 2023). However, whether DGUOK-AS1 can promote EGFR-TKI resistance still needs to be further investigated. This review focuses on the mechanisms of lncRNAs in EGFR-TKI resistance in NSCLC, but in fact, circRNAs, a subclass of lncRNAs, are also involved in regulating EGFR-TKI resistance (Zhou et al., 2019; Huang Y. et al., 2020; Ma et al., 2020; Yang et al., 2021; Dai et al., 2022; Fan et al., 2022; Niu et al., 2022; Wang et al., 2022; Pan et al., 2023; Wen et al., 2023). The relevant circRNAs are listed in Table 2.

**TABLE 2:** CircRNAs involved in EGFR-TKI resistance of NSCLC.

circRNA	Expression	Target/Signaling network	Function	Reference
hsa_circ_0004015	↑	miR-1183/PDPK1	gefitinib resistance	Zhou et al. (2019)
hsa_circ_0002130	↑	miR-498	osimertinib resistance	Ma et al. (2020)
circSETD3	↑	miR-520h/ABCG2	gefitinib resistance	Huang et al. (2020b)
circSETD3	↑	FXR1/ECT2	gefitinib resistance	Wen et al. (2023)
circRNA_102481	↑	miR-30a-5p/ROR1	EGFR-TKI resistance	Yang et al. (2021)
circ_0014235	↑	miR-146b-5p/YAP/PD-L1	gefitinib resistance	Niu et al. (2022)
circ_MACF1	↓	miR-942-5p/TGFBR2	gefitinib resistance	Fan et al. (2022)
hsa_circ_0000567	↑	miR-377-3p/ZFX	gefitinib resistance	Wang et al. (2022)
hsa_circ_0007312	↑	miR-764/MAPK1	osimertinib resistance	Dai et al. (2022)
circRBM33	↑	DNMT1/IL-6	osimertinib resistance	Pan et al. (2023)

## 8 Conclusion and perspective

In recent years, EGFR-TKI treatment for NSCLC has shown promising efficacy, but the development of drug resistance is unavoidable and significantly limits the therapeutic potency of EGFR-TKI treatments (Mok et al., 2009; Zhou et al., 2011). Therefore, in order to overcome EGFR-TKI resistance and improve patients’ prognosis, researchers have conducted numerous in-depth investigations into the mechanism of EGFR-TKI resistance. Due to the rapid development of RNA sequencing technology and bioinformatics analysis, many lncRNAs were found to participate in the regulation of EGFR-TKI resistance in NSCLC (Cheng et al., 2015a; Shi et al., 2020). The evidence described in this review suggests that lncRNAs can influence EGFR-TKI resistance by regulating aberrant activation of signaling pathways, phenotypic transformation, tumor microenvironment, and other modalities.

However, the research of lncRNAs in EGFR TKI-resistant NSCLC is still in its infancy, and some limitations exist. To start with, most studies concentrated on the downstream mechanisms underlying dysregulated lncRNAs in EGFR TKI-resistant NSCLC, while the upstream mechanism of dysregulation of lncRNA is unclear. Second, there are many lncRNAs that have been demonstrated to be implicated in NSCLC progression, but their relationship with EGFR-TKI resistance was not further explored (Lv et al., 2021; Xu et al., 2021). In third place, despite the overwhelming evidence that lncRNAs have a crucial role in EGFR-TKI resistance, to date, lncRNAs have not been clinically validated for their potential value. In terms of diagnosis and prediction, exosomal lncRNAs have great clinical application value and may be the focus of clinical application exploration due to their natural advantages of wide distribution and easy access to materials (Tao et al., 2020; Han et al., 2021). On the therapeutic side, lncRNAs targeting therapies, which rely on technologies such as RNAi, antisense oligonucleotides (ASOs), adenoviral/lentiviral vectors and CRISPR-Cas9, face many challenges (Goyal et al., 2017; Verdera et al., 2020; Kara et al., 2022), particularly safety concerns caused by off-target effects, which still need to be overcome (Toden et al., 2021). Intriguingly, many studies have demonstrated that certain drugs can regulate lncRNAs to reverse EGFR-TKI resistance in NSCLC by targeting upstream or downstream molecules of lncRNAs. Examples include the upregulation of GAS5 by ecto-ATP synthase inhibitor (Chang et al., 2020), the downregulation of MALAT1 by polyphylin I (Yang et al., 2018), the downregulation of HOTAIR by berberine (Zheng F. et al., 2020), and the downregulation of CCAT1 by hyperoside (Hu et al., 2020), all of which shed fresh light on dealing with EGFR-TKI resistance, that is, targeting upstream or downstream molecules of lncRNAs is also a possible alternative therapeutic approach.

In conclusion, lncRNAs are crucial in EGFR-TKI resistance of NSCLC via a variety of mechanisms. More in-depth investigations are warranted to further uncover the regulatory role of lncRNAs in EGFR-TKI resistance and apply lncRNAs to clinical practices to promote the prognosis of NSCLC patients.
